# Ethnic Differences in Cardiometabolic Risk Profile at Age 5–6 Years: The ABCD Study

**DOI:** 10.1371/journal.pone.0043667

**Published:** 2012-08-20

**Authors:** Marieke L. A. de Hoog, Manon van Eijsden, Karien Stronks, Reinoud J. B. J. Gemke, Tanja G. M. Vrijkotte

**Affiliations:** 1 Department of Public Health, Academic Medical Centre, University of Amsterdam, Amsterdam, The Netherlands; 2 Department of Epidemiology, Documentation and Health Promotion, Public Health Service, Amsterdam, The Netherlands; 3 Institute of Health Sciences, VU University, Amsterdam, The Netherlands; 4 Department of Paediatrics, EMGO institute, Institute of Cardiovascular Research, VU University Medical Centre, Amsterdam, The Netherlands; Erasmus Medical Center, The Netherlands

## Abstract

**Background:**

To examine ethnic differences in cardiometabolic risk profile in early age, and explore whether such differences can be explained by differences in body mass index (BMI) or waist circumference (WC).

**Method:**

Anthropometric measurements, blood pressure and (in a subsample) fasting blood were collected during a health check of 2,509 children aged 5–6 years. Four ethnic groups were distinguished: Dutch (n = 2,008; blood n = 1,300), African descent (n = 199; blood n = 105), Turkish (n = 108; blood n = 57) and Moroccan (n = 194; blood n = 94). Ethnic differences in diastolic and systolic blood pressure (DBP/SBP), fasting glucose, low-density lipoprotein (LDL), high-density lipoprotein (HDL) and triglyceride levels were determined and the explanatory role of BMI and WC was examined with regression analysis.

**Results:**

After adjustment for confounders, African descent children showed higher DBP (β2.22 mmHg; 95%CI:1.09–3.36) and HDL levels (β:0.09 mmol/l; 95%CI:0.03–0.16) compared to Dutch children (reference group). Turkish children showed higher SBP (β:1.89 mmHg; 95%CI:0.25–3.54), DBP (β:2.62 mmHg; 95%CI:1.11–4.13), glucose (β:0.12 mmol/L; 95%CI:0.00–0.25) and triglyceride levels (β:0.13 mmol/L; 95%CI:0.02–0.25). Higher BMI values were found in all non–Dutch groups (differences ranged from 0.53–1.03 kg/m^2^) and higher WC in Turkish (β:1.68 cm; 95%CI:0.99–2.38) and Moroccan (β:1.65 cm; 95%CI:1.11–2.19) children. BMI and WC partly explained the higher SBP/DBP and triglyceride levels in Turkish children.

**Conclusion:**

Ethnic differences in cardiometabolic profile exist early in life and are partly explained by differences in BMI and WC. African children showed favourable HDL levels and Turkish children the most unfavourable overall profile, whereas their Moroccan peers have less increased cardiometabolic risk in spite of their high BMI and WC.

## Introduction

Results of studies examining ethnic differences in risk of cardiovascular disease (CVD) and diabetes type 2 (DM2) are varied and sometimes conflicting. Overall, in Western countries (e.g. the USA/UK), cardiovascular mortality seems to be generally higher among ethnic minority groups compared to the host population [1]–[4]. The Netherlands is no exception to this; although the cardiovascular mortality among Moroccan tends to be lower [5]–[7]. Looking at the individual components of the cardiometabolic risk factors, African people, for example, tend to have healthier (higher) high-density lipoprotein (HDL) and lower triglyceride levels, but are more likely to be hypertensive than whites [8], [9]. The prevalence of hypercholesterolemia is lower among Turkish and Moroccan adults living in the Netherlands [7], [10]. However, part of this advantage is off-set by their relatively low HDL cholesterol, resulting in an unfavourable total/HDL cholesterol ratio, particularly in the Turkish population [7]. Paradoxically, the risk of developing DM2 and obesity is reported to be higher among all ethnic minority groups [6], [10]–[12].

Overweight is seen as an important trigger for developing DM2, hyperlipidaemia and hypertension [13]–[16]. Because overweight is more common in some ethnic minority groups, this might explain the ethnic differences in cardiometabolic risk profile.

Early-life exposures have been implicated in the aetiology of CVD, and the presence of cardiometabolic risk factors during childhood persist into adulthood [17]–[19]. Therefore, knowledge on early emergence of ethnic differences in cardiometabolic risk factors is important. Studies on this subject among several minority groups reported that these differences in childhood and adolescence generally follow adult patterns [20]–[24]. However, in most of these studies the children were aged ≥8 years or were adolescents (14–16 years). To our knowledge only one study, investigating an overweight and obese cohort (3–18 years), reported higher prevalence of cardiometabolic risk factors (e.g. low HDL-cholesterol, high triglycerides and impaired glucose tolerance) among overweight Turkish children compared to their Dutch and Moroccan overweight peers [25]. Data on healthy population-based measures in these particular groups are missing.

To gain additional knowledge, the present study was designed to examine ethnic differences in cardiometabolic risk profile in healthy children aged 5–6 years of Dutch, African descent,Turkish and Moroccan origin; the main ethnic groups living in the Netherlands. Furthermore, we determined to what extent body mass index (BMI; as a measure of total body fat) and waist circumference (WC; as a measure of abdominal fat) affect any observed differences. Since African descent children and particularly children from Turkish and Moroccan origin are consistently found to have high prevalence rates of overweight [26]–[29], and adiposity measures are correlated with cardiometabolic risk [30], [31], we hypothesized that BMI and WC would (partly) explain the ethnic difference in cardiometabolic risk profile.

## Methods

### Ethics Statement

The present study is part of the Amsterdam Born Children and their Development (ABCD) study. The main goal of this study is to examine and determine factors in early life (during pregnancy and infancy) that might explain the later health of the child with specific attention paid to ethnic inequalities. Approval was obtained from the Academic Medical Center Medical Ethical Committee, the VU University Medical Center Medical Committee and the Registration Committee of Amsterdam. All participating mothers gave written informed consent for themselves and their children.

### Subjects

The design and rationale of the ABCD study have been described previously [32]. In brief, between January 2003 and March 2004 8,266 pregnant women were included in the study after their first antenatal visit to an obstetric caregiver (phase 1). They filled out an extensive pregnancy questionnaire about socio-demographic data, obstetric history, lifestyle, dietary habits, and psychosocial conditions. Of these respondents, 7,863 women gave birth to a viable singleton infant and 6,575 women gave permission to collect information obtained from the Youth Health Care (phase 2). Phase 3 of the study started in the summer of 2008. Around two weeks after their ABCD-child’s fifth birthday, 6,161 mothers who initially gave permission for follow-up (93.7% of 6,575) were sent a questionnaire (in Dutch, English or Turkish) in which they were also asked for permission on participation of their child in the physical examination and blood collection. Reasons for lack of follow-up included withdrawal from the study, infant or maternal death, and loss to follow-up due to unknown address or emigration. The questionnaire, returned by 4,488 mothers, provided information on their child’s health, development and behaviour. Various physical measurements, including height, bodyweight, WC and blood pressure (BP), took place in 3,321 children (taking about 1 h per child). In a subsample of 2,003 (60.3% of 3,321) children, whose parents gave additional permission apart from the physical examination, fasting blood was also collected (fasting plasma glucose (mmol/L), low-density lipoprotein (LDL; mmol/L), HDL (mmol/L), and triglyceride (mmol/L).

The present study includes 2,509 singleton children (aged 5–6 years) from the main ethnic groups living in the Netherlands (Dutch, African descent, Turkish and Moroccan origin), for which the 5-year questionnaire and the physical examination, was completed. Of these children 1,556 (62.0% of 2,509) had known fasting blood values (fasting glucose, and various lipid levels). Ethnicity of the child was based on the country of birth of the child’s mother and her mother (self reported) in order to include children from both first-generation (born outside the Netherlands) and second-generation (born in the Netherlands, but with a grandmother born in another country) mothers. Because they have a similar ethnic background (all originally from sub-Saharan Africa) [5], children from Surinam (Surinam-Creole), the Antilles, Ghana and other Sub-Saharan African origin were combined in the ‘African descent’ group.

The ethnic groups were composed as follows: Dutch (n = 2,008; for blood values n = 1,300) African descent (n = 199; for blood values n = 105), Turkish (n = 108; for blood values n = 57) and Moroccan (n = 194; for blood values n = 94). Children from ethnic groups other than those mentioned above (n = 812; for blood values n = 447) were excluded, because their numbers would be too small to analyze separately.

Comparison of the included 1,556 participants with measured fasting blood with the 953 (2,509–1,556) participants without fasting blood measurement, showed a higher proportion of Dutch ethnicity (83.5% vs. 74.3%; *p*<0.001) and lower systolic BP (SBP; 97.4 vs. 98.6 mmHg; *p* = 0.001) and diastolic BP (DBP; 57.4 vs. 58.8 mmHg; *p*<0.001) but no difference on mean BMI and WC.

### Measurements

The outcome variables measured were: BP expressed in mmHg (SBP and DBP), fasting plasma glucose (mmol/L), LDL (mmol/L), HDL (mmol/L), and triglyceride (mmol/L). For BP, first a test measure was made (to comfort/relax) the child followed by a 10-min rest period. The device used was the Omron 705 IT (Omron Healthcare Inc, Bannockburn, IL, USA) with its appropriate cuff size (arm circumference 17–22 cm). Then, BP was measured twice on the right arm in sitting position, with the arm supported at heart level. These two measurements were considered valid if they did not differ by more than 10 mmHg, otherwise BP was measured a third time. SBP and DBP (mmHg) were calculated by taking the mean value of the two valid measures. Capillary blood was taken in the morning between 7:45 and 8:30 by finger prick collected in a validated kit developed for ambulatory purposes (LabAnywhere, Haarlem, the Netherlands) [33].

For the present study the variables of body composition were: BMI (kg/m^2^) and WC (cm). To calculate BMI, height was measured to the nearest millimeter using a Leicester portable height measure (Seca), and weight to the nearest 100 gram using a Marsden weighing scale, (model MS-4102). WC was measured midway between the costal border and the iliac crest to the nearest millimeter using a Seca measuring tape. Overweight/obesity was defined by the age- and sex specific International Obesity Task Force (IOTF) guidelines [34].

### Data Analysis

Demographic ethnic differences between anthropometrics and cardiometabolic risk factors were examined with χ^2^-tests (categorical data) or ANOVA (continuous data). Linear regression was used to examine ethnic differences in SBP and DBP, glucose, LDL, HDL and triglyceride levels, and the role of BMI and WC in explaining these differences. In order to be able to research the explanatory role of BMI and WC for ethnic differences in blood pressure, glucose and cholesterol levels these variables should be associated with these outcome variables in general. Therefore first, the associations of BMI and WC with these cardiometabolic risk factors were assessed with linear regression after adjustment for age, sex, and ethnicity. Because SBP, DBP and WC are associated with height, all models including these variables were additionally adjusted for child’s height. Second, to detect any ethnic differences in BMI, WC, SBP, DBP, glucose, LDL, HDL and triglyceride levels analysis were performed with adjustment for age, sex and, where appropriate, additionally for height (model 1). Third, for only those cardiometabolic risk factors in which ethnic differences were present in model 1 (Dutch  =  reference group), as well as having an association with BMI or WC in general, the explanatory role of BMI or WC was assessed in two separate models by adding BMI or WC (including height) to model 1.

To provide an adequate fit, all models were tested for possible non-linearity. Interactions between BMI and WC with ethnicity were tested to reveal possible ethnic-specific associations. Restricted cubic splines (RCS) were used in the regression analysis to examine and characterize an association that is suspected to be non-linear by using higher order piecewise polynomials to accommodate potential changes in the direction of the association across the exposure distribution [35]. If the results for a given model indicated that a linear model provided an adequate fit (likelihood ratio test: p-value >0.05), we report the results from a linear model.

Because fasting triglyceride concentrations were positively skewed, we used log values in regression analyses; the distributions and the residuals in the regression models became normal with these transformations.

Statistical analyses were conducted using *R* 2.12.1. A p-value <0.05 was regarded as statistically significant.

## Results

Table 1 presents the characteristics of the children at age 5–6 years, stratified for ethnicity. Dutch children were on average younger (p = 0.01), but there were no differences between the ethnic groups with respect to percentage boys and girls. The percentage overweight/obese was highest among Turkish (27.0%) and Moroccan (26.2%) children (Dutch 6.6%). Ethnic differences in metabolic parameters, adjusted for age and sex are described below.

**Table 1:** Characteristics of the study sample stratified for ethnicity.

	Dutch n = 2008 (n = 1300 for blood)	African descent n = 199 (n = 105 for blood)	Turkish n = 108 (n = 57 for blood)	Moroccan n = 194 (n = 94 for blood)
	Mean (SD) or %			
Age (years)	5.7 (0.5)	5.9 (0.5)	5.9 (0.5)	6.1 (0.6)
Sex % boys	50.8	47.1	52.0	56.7
Height (cm)	116.9 (5.7)	119.1 (6.0)	116.7 (5.3)	118.1 (6.4)
Weight (kg)	21.0 (2.9)	22.8 (4.3)	22.5 (3.8)	23.1 (4.3)
BMI (kg/m^2^)	15.4 (1.3)	16.0 (2.0)	16.5 (2.0)	16.5 (1.9)
Waist circumference (cm)	52.3 (3.3)	53.2 (4.8)	54.4 (4.7)	54.5 (4.7)
Overweight/obese %	6.6	17.8	27.0	26.2
Systolic BP (mmHg)	97.5 (8.5)	99.0 (8.9)	99.5 (8.8)	99.8 (9.3)
Diastolic BP (mmHg)	57.4 (7.8)	60.4 (7.4)	60.4 (8.5)	59.5 (7.6)
Glucose (mmol/L)	4.6 (0.5)	4.5 (0.5)	4.7 (0.4)	4.6 (0.5)
LDL cholesterol (mmol/L)	2.3 (0.6)	2.4 (0.7)	2.4 (0.6)	2.3 (0.6)
HDL cholesterol (mmol/L)	1.30 (0.31)	1.40 (0.34)	1.35 (0.26)	1.30 (0.26)
Triglyceride (mmol/L)	0.64 (0.30)	0.64 (0.33)	0.75 (0.45)	0.64 (0.26)

### Association between Body Composition and Cardiometabolic Risk Factors

**Figure 1:**
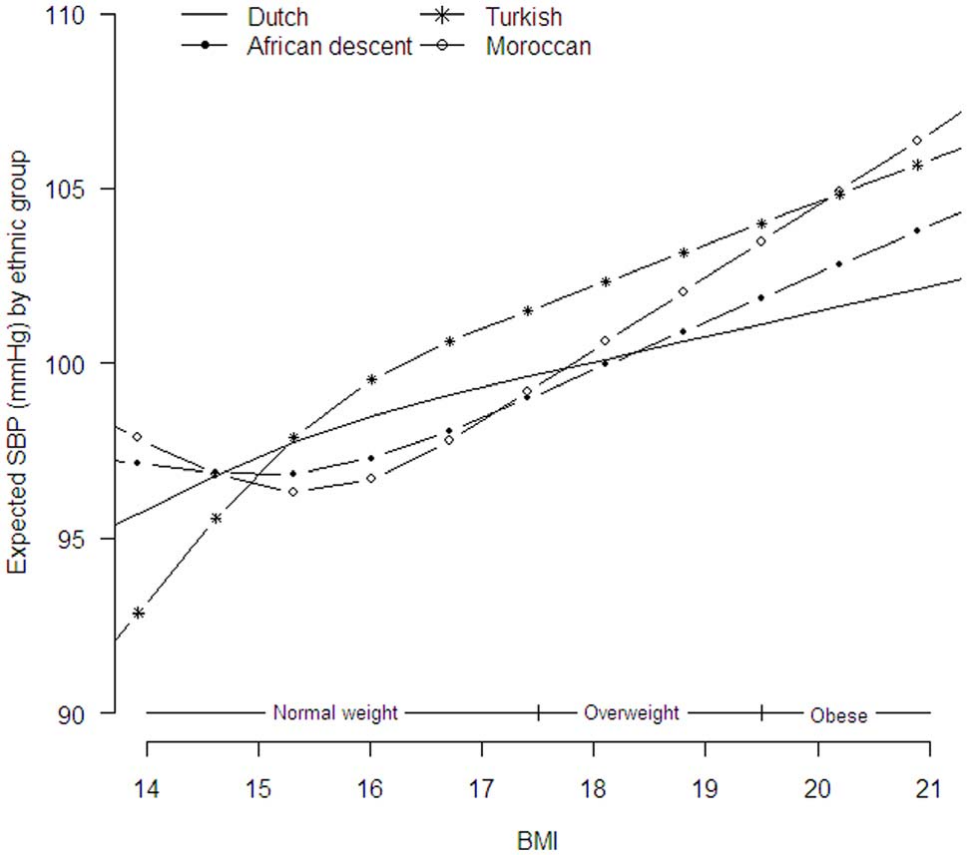
Estimated expected SBP (mmHg) per ethnic group, as a function of the child’s BMI. Overweight status cut off scores based on boys with mean age 5.5 years [34].

Table 2 presents overall associations between the cardiometabolic risk factors and BMI or WC adjusted for age, sex, height (for SBP, DBP and WC) and ethnicity. BMI was non-linear and showed an ethnic-specific association with SBP (likelihood ratio test p = 0.02; Figure 1). Figure 1 showed a steeper increase in SBP with increasing BMI in Turkish (across the whole BMI range) and Moroccan (in the mid normal and overweight BMI range) children compared to the ethnic Dutch children. BMI was linear, not ethnic-specific associated with DBP and glucose; one unit increase in BMI (kg/m^2^) resulted in 1.24 mmHg increase in DBP (95%CI: 0.88, 1.60) and a small increase of 0.05 mmol/L in glucose levels (95%CI: 0.02, 0.08; Table 2). No associations were found between BMI and LDL, HDL and triglyceride.

**Table 2:** Associations of body mass index (BMI) and waist circumference (WC) with cardiometabolic risk factors.

	Systolic BP (mmHg)	Diastolic BP (mmHg)	Glucose (mmol/L)	LDL cholesterol (mmol/L)	HDL cholestrol (mmol/L)	(log)triglyceride (mmol/L)
BMI (kg/m^2^)	**2.01 (1.50,2.52)** * †	**1.24 (0.88, 1.60)**	**0.05 (0.02, 0.08)**	0.01 (−0.02, 0.05)	0.00 (−0.02, 0.02)	0.01 (−0.01, 0.04)
WC (cm)	**1.48 (1.04, 1.92)**	**1.02 (0.61, 1.42)**	0.02 (−0.01, 0.05)	**0.04 (0.00, 0.09)**	−0.02 (−0.04, 0.00)	**0.05 (0.02, 0.08)**

Beta’s are adjusted for age, sex and ethnicity; models including SBP, DBP or WC are additionally adjusted for height.

* Bold indicates significant different from reference group;

† Non-linear association: estimated betas for mean BMI (15.3 kg/m^2^).

All associations with WC were linear and similar for all ethnic groups (likelihood ratio test p<0.10). One unit increase in WC (cm) was associated with an increase of 1.48 mmHg in SBP (95%CI: 1.04, 1.92), and 1.02 mmHg in DBP (95%CI: 0.61, 1.42; Table 2). Furthermore, one unit increase in WC was also associated with a small increase of 0.04 mmol/L in LDL (95%CI: 0.00, 0.09) and 0.05 mmol/L in triglyceride levels (95%CI: 0.02, 0.08). No association was found between WC, glucose and HDL.

The correlation between BMI and WC was found to be strong (r = 0.77; p-value <0.001).

### Ethnic Differences in Cardiometabolic Risk Profile

Table 3 presents data on ethnic differences in cardiometabolic risk factors after adjustment for age and sex. Compared with the ethnic Dutch children (reference group), African descent children showed a higher BMI (β: 0.53 kg/m^2^; 95%CI: 0.32–0.74), DBP (β: 2.22 mmHg; 95%CI: 1.09–3.36) and HDL levels (β: 0.09 mmol/L; 95%CI: 0.03–0.16). The Turkish children showed a higher BMI (β: 1.02 kg/m^2^; 95%CI: 0.75–1.30), WC (β: 1.68 cm; 95%CI: 0.99–2.38), SBP (β: 1.89 mmHg; 95%CI: 0.25–3.54), DBP (β: 2.62 mmHg; 95%CI: 1.11–4.13), glucose levels (β: 0.12 mmol/L; 95%CI: 0.00–0.25) and triglyceride levels (β: 0.13 mmol/L; 95%CI: 0.02–0.25). The Moroccan children showed only higher BMI (β: 1.03 km/m^2^; 95%CI: 0.81–1.24) and WC (β: 1.65 cm; 95%CI: 1.11–2.19) compared to the ethnic Dutch children.

**Table 3:** Ethnic differences in cardiometabolic risk factors (ethnic Dutch  =  reference group) and the effect of additional adjustment for body mass index (BMI) or waist circumference (WC).

		African descent	Turkish	Moroccan	
	Adjusted?	Difference (95%CI)			
BMI (kg/m^2^)	Model 1	**0.53 (0.32, 0.74)** *	**1.02 (0.75, 1.30)**		**1.03 (0.81, 1.24)**
Waist circumference (cm)	Model 1	0.47 (−0.06, 1.00)	**1.68 (0.99, 2.38)**		**1.65 (1.11, 2.19)**
Systolic BP (mmHg)	Model 1	0.34 (−0.89, 1.57)	**1.89 (0.25, 3.54)**		0.98 (−0.28, 2.23)
	BMI†	–$	0.17 (−1.81, 2.15)		–
	WC	–	1.11 (−0.53, 2.76)		–
Diastolic BP (mmHg)	Model 1	**2.22 (1.09, 3.36)**	**2.62 (1.11, 4.13)**		1.13 (−0.04, 2.29)
	BMI	**1.83 (0.70, 2.96)**	**1.76 (0.24, 3. 28)**		–
	WC	**2.14 (1.01, 3.27)**	**2.09 (0.57, 3.61)**		–
Glucose (mmol/L)	Model 1	−0.03 (−0.13, 0.06)	**0.12 (0.00, 0.25)**		0.01 (−0.09, 0.12)
	BMI	–	0.10 (−0.03, 0.23)		–
	WC	–	–		–
LDL cholesterol (mmol/L)	Model 1	0.04 (−0.08, 0.17)	0.09 (−0.08, 0.25)		−0.03 (−0.18, 0.09)
	BMI	–	–		–
	WC	–	–		–
HDL cholesterol (mmol/L)	Model 1	**0.09 (0.03, 0.16)**	0.04 (−0.04, 0.12)		−0.02 (−0.09, 0.04)
	BMI	**–**	–		–
	WC	**–**			
(log)triglyceride (mmol/L)	Model 1	−0,02 (−0.11, 0.06)	**0.13 (0.02, 0.25)**		0.01 (−0.08, 0.10)
	BMI	–	**–**		–
	WC	–	0.06 (−0.01, 0.22)		–

Model 1: Adjusted for age and sex; models including SBP, DBP or WC are additionally adjusted for height.

* Bold indicates significant different from reference group;

† Non-linear association: estimated beta for mean BMI (15.3 kg/m^2^);

$ - No ethnic differences present in model 1 and/or association with BMI/WC (Table 2).

### Explanatory Role of Body Composition for Ethnic Differences in Cardiometabolic Risk Profile

**Figure 2:**
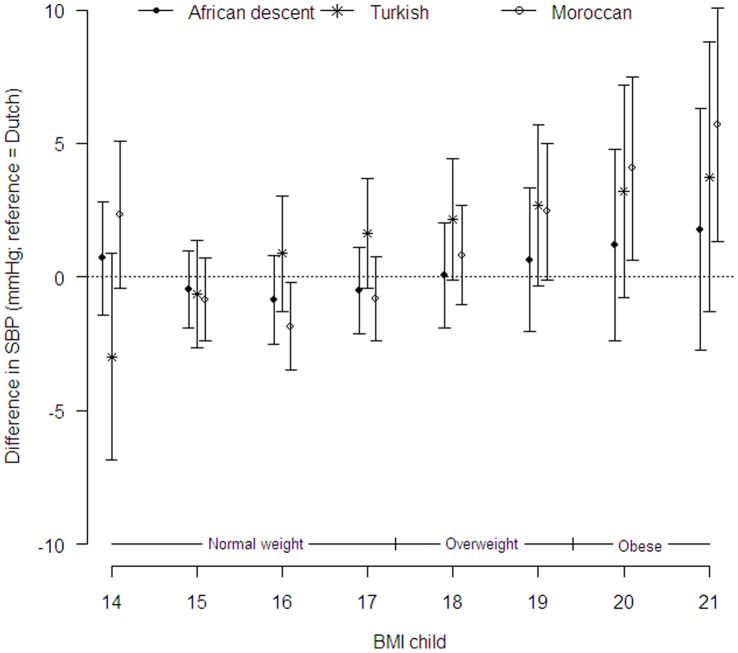
Difference in SBP (mmHg) per ethnic group (Dutch  =  reference group), as a function of the child’s BMI. Overweight status cut off scores based on boys with mean age 5.5 years [34].

Adjustment for BMI had little effect on the size of glucose and blood lipid differences in all ethnic groups (Table 3). There was a strong effect of BMI on DBP (linear, not ethnic specific) and SBP (non-linear, ethnic specific (likelihood ratio test p = 0.02)). Figure 2 shows that the differences in SBP for the Turkish children are attenuated after adjustment for BMI. The differences in SBP in Turkish children became non-significant across the whole BMI range. Although no mean differences in SBP were found in the Moroccan children in model 1 (table 3), after adjustment for BMI obese Moroccan children showed higher SBP compared to the Dutch children (figure 2). The difference in DBP in African descent and Turkish children became smaller after adjustment for BMI. However, DBP remained 1.83 mmHg (95%CI: 0.709–2.96) higher in African descent and 1.76 mmHg (95%CI: 0.24–3.28) higher in Turkish children compared to the Dutch children.

Adjustment for WC showed results comparable to BMI, although WC explained the ethnic differences to a lesser degree, except for the triglyceride levels in Turkish children. After additional adjustment for WC, the higher triglyceride levels in Turkish children attenuated. Because Moroccan children did not differ in blood pressure, glucose, LDL, HDL and triglyceride level from the ethnic Dutch children in model 1, the explanatory role of BMI and WC was not tested (Table 3).

## Discussion

This study examined ethnic differences in cardiometabolic risk profile, and the explanatory role of BMI and WC in a healthy population of Dutch, African descent, Turkish and Moroccan 5–6 year olds. We found that ethnic differences in cardiometabolic risk profile do exist at young age. All non-Dutch groups showed higher BMI and WC values compared to the Dutch children, except for WC in the African children. After adjustment for confounders (age, sex and height), African descent children showed higher DBP levels and more favourable HDL levels, and Turkish children higher SBP and DBP and somewhat higher glucose and triglyceride levels. On the other hand, the Moroccan children (except for the higher BMI and WC) showed hardly any differences in cardiometabolic risk profile. BMI and WC partly explained the ethnic difference in BP, glucose and triglyceride levels, but ethnic differences remained for DBP (African descent and Turkish).

Ethnic differences in cardiometabolic risk profile have been reported in both adults and children [9], [20], [22], [24], [25], [36], [37], with lower rates of CVD in Africans (especially men) and Moroccans and higher rates among Turkish groups compared to the host population. A Dutch study among overweight/obese Turkish and Moroccan children showed significantly higher rates of cardiometabolic risk in Turkish children relative to their Moroccan and ethnic Dutch peers [25]. The present data support the notion that Turkish children have a more adverse cardiometabolic risk profile compared to Moroccan and ethnic Dutch children. Children in Turkish cohorts appear to have high mean glucose and triglyceride levels, but also high HDL-cholesterol. Probably immigration of Turkish children to the Netherlands has an additional adverse effect on cardiometabolic risk profile [38].

Adiposity is seen as an important factor in explaining cardiometabolic outcomes [15], [39] and we found higher rates of overweight in all ethnic minority groups. Unexpectedly, the associations between BMI and WC, and the different blood measures, were weak or even absent. There is evidence that, at least in adults, visceral fat promotes systemic inflammation by secreting inflammatory acute phase proteins, resulting in a higher risk of developing metabolic diseases, especially diabetes [40], [41]. Possibly, this mechanism may not be present at such a young age [42], or the variations in BMI, WC or the blood chemistry values were too small. In addition, BMI and WC were highly correlated (r = 0.77) which might explain why data didn’t show much differences in the association between BMI and WC with the other cardiometabolic risk factors. This suggests that the distinctiveness between overall and abdominal fat is low in young children.

After adjustment for BMI and WC the DBP of Turkish and African descent children remained higher. Furthermore, Turkish and Moroccan infants showed a steeper increase in SBP with increasing BMI resulting in a higher SBP in obese Moroccan children. This implies that other mechanisms also influence blood pressure in these children. Rapid growth velocity during infancy is seen as a risk factor for elevated blood pressure [43]. Previous findings of the ABCD study showed that rapid growth in the first 6 months is more common in infants of Turkish, Moroccan and African origin [44], Ujcic-Voortman et al. reported that Turkish adults have higher C-reactive protein levels [45], a marker of inflammation associated with elevated BMI [46] and increased risk of coronary heart diseases [47]. Although causation is hard to prove, hypothetically, this might have its origin in early life and already influence BP in Turkish children; still, more research is needed to substantiate this hypothesis. In addition, the higher DBP in the African descent children might be related to the renin-angiotensin-aldosteron system and potassium balance [48]. There is evidence that children from African-American origin have a lower urinary excretion rate of potassium, which makes them predisposed to hypertension [48]. This might be due to differences in diet such as high sodium and low potassium intake and hereditary predisposition [48]–[50].

Despite higher BMI levels, African adults and children tend to have a healthier lipid profile. Higher levels of HDL are often found in both adults and children from African descent [9], [20], [24]. For example, the Chase Study of Whincup et al. (which included black African-Caribbean children aged 9–10 years) found higher HDL levels in this group compared to white Europeans (% difference HDL: 1.9; 95% CI: 0.2–3.6) after adjustment for age and sex [24]. Although this might indicate a genetic component, the mechanisms responsible for these differences are not yet elucidated. However, there is evidence that the -514T hepatic lipase allele, which is associated with low hepatic lipase activity and increased HDL concentration, is more common among African people [51].

The higher HDL levels in Africans are often accompanied by lower triglyceride levels [9], [24], [52]. The combination of higher HDL levels and lower triglyceride levels suggest that African people may have a different deposition of fat. Less abdominal fat, frequently described in Africans [52]–[54], is associated with decreased adipose cell basal lipolysis, resulting in higher HDL and lower triglyceride levels [9]. Although our study did not show lower triglyceride levels in the African descent group, these differences may become more visible in adolescent or adult age. Moreover, we need to bear in mind that the ‘African descent’ group is composed of all those from sub-Saharan African origin (including Surinamese-Creole and Antilles) which makes it a heterogeneous group. Although these children have a comparable ethnic background, the cardiometabolic risk profiles may differ between these smaller groups. Nevertheless, sub-analyses indicate no differences between Black Africans (sub-Saharan) and Black Caribbean’s (Surinam-Creole and Antilles) in all outcome variables (results not shown).

It is well known that overweight in childhood tracks into adulthood [55] and there is also evidence that the same accounts for blood pressure and lipid levels [56], [57]. As we shown in this study overweight is highest in Turkish and Moroccan children and has already its consequences in Turkish children. As a result of the high prevalence of overweight in Moroccan children, the currently lower CVD rates in Moroccan adults might converge towards the prevalence rates of ethnic Dutch adults, or beyond when these children become adults. This implies that preventing high levels of overweight and other cardiometabolic risk factors during childhood might decrease risk of CVD later in life.

To our knowledge, this is the first study to examine ethnic differences in cardiometabolic risk profile in a European cohort including population-based Turkish and Moroccan children. When interpreting our results, some limitations should be taken into account. First, only a subsample of children had blood taken. Due to small groups, the lack of significant differences may be due to lack of power. Alternatively, we might have underestimated the ethnic differences in cardiometabolic risk due to selection of the group with fasting blood measures. The children with fasting blood measures had a lower mean BP, which suggests a relative healthier group compared to the children without fasting blood measures. However, no differences were found in mean BMI and WC. Second, previous studies showed sex differences in adiposity measures, BP and lipid levels [54], [58]. Due to small groups we weren’t able to stratify our results by sex. In addition, we found no evidence to stratify by sex; likelihood ratio test did not show any ethnic specific associations with sex for all outcome measures (results not shown). We only adjusted for sex as a confounder. Third, measuring BP in children is more difficult than in adults, e.g. readings are likely to be falsely high in children who find it difficult to relax their arm during the measurement. To control for falsely high or low blood pressures, the BP measurements were performed according to a standardized protocol which included a test measurement before actual measurements were taken [59]. Finally, WC is difficult to measure. Due to lack of a standardized technique to measure WC in children, caution is required when comparing WC between studies. Especially in overweight children, variability in measurement may increase with higher levels of body fat [60]. However, WC as measured in the present study represents the measurement site most closely associated with cardiometabolic risk factors in overweight children [61].

In conclusion, there are ethnic differences in cardiometabolic risk profile at the age of 5–6 years, with favourable HDL levels in African descent children and Turkish children showing the most adverse profile. Moroccan peers have less increased cardiometabolic risk in spite of their high BMI and WC. Although the associations between BMI and WC with the other cardiometabolic risk factors were weak; the ethnic differences in blood pressure, glucose and triglyceride level appeared to be partly accounted for by ethnic differences in adiposity. Future studies should focus on other explanatory factors such as early rapid growth during infancy [43], specific diet components (e.g. sodium and potassium intake) [49] or inflammatory markers [45]. Longitudinal research is needed to determine whether these findings lead to a higher risk of CVD in adult age in the different ethnic groups. Although not all differences are explained by the higher adiposity rates, weight management in children is important to prevent ethnic disparities in cardiometabolic function during childhood and later in life.
